# The early detection of immunoglobulins via optical-based lateral flow immunoassay platform in COVID-19 pandemic

**DOI:** 10.1371/journal.pone.0254486

**Published:** 2021-07-20

**Authors:** Pang-Yen Chen, Cheng-Hao Ko, C. Jason Wang, Chien-Wei Chen, Wei-Huai Chiu, Chitsung Hong, Hao-Min Cheng, I-Jen Wang

**Affiliations:** 1 Department of Emergency Medicine, Mackay Memorial Hospital, Taipei, Taiwan; 2 Institute of Environmental and Occupational Health Sciences, National Yang Ming Chiao Tung University College of Medicine, Taipei, Taiwan; 3 Institute of Public Health, National Yang Ming Chiao Tung University College of Medicine, Taipei, Taiwan; 4 SpectroChip Inc., Hsinchu, Taiwan; 5 Graduate Institute of Automation and Control, National Taiwan University of Science and Technology, Taipei, Taiwan; 6 Department of Pediatrics and Medicine, Stanford University School of Medicine, Stanford, California, United States of America; 7 Department of Diagnostic Radiology, Chang Gung Memorial Hospital Chiayi Branch, Chiayi, Taiwan; 8 College of Medicine, Chang Gung University, Taoyuan, Taiwan; 9 Chung Shan Medical University, Taichung, Taiwan; 10 Department of Medicine, National Yang Ming Chiao Tung University College of Medicine, Taipei, Taiwan; 11 Center for Evidence-based Medicine, Taipei Veterans General Hospital, Taipei, Taiwan; 12 Department of Medical Education, Taipei Veterans General Hospital, Taipei, Taiwan; 13 Department of Pediatrics, Taipei Hospital, Ministry of Health and Welfare, Taipei, Taiwan; 14 College of Public Health, China Medical University, Taichung, Taiwan; 15 National Institute of Environmental Health Sciences, National Health Research Institutes, Miaoli, Taiwan; 16 National Taiwan University Hospital, National Taiwan University, Taipei, Taiwan; Waseda University: Waseda Daigaku, JAPAN

## Abstract

The coronavirus disease (COVID-19) is the global public health challenge currently persisting at a grand scale. A method that meets the rapid quantitative detection of antibodies to assess the body’s immune response from natural COVID-19 illness or vaccines’ effects is urgently needed. In the present study, an attempt was made to integrate a newly designed spectrometer to the COVID-19 test strip procedure; this augmentation provides the quantitative capacity to a lateral flow immunoassay (LFIA). Optical interpretation of results by quantitative α index, rather than visual qualification, can be done quickly, in 5–10 minutes. The developed product was compared with several other serological IgM/IgG antibody reagents on the market by recruiting 111 participants suspected of having COVID-19 infection from March to May 2020 in a hospital. Taking RT-PCR as the diagnostic gold standard, the quantitative spectral LIFA platform could correctly detect all 12 COVID-19 patients. Concerning RT-PCR negative patients, all three antibody testing methods found positive cases. The optical-based platform exhibited the ability of early detection of immunoglobulins of RT-PCR negative patients. There was an apparent trend that elevation of IgM levels in the acute phase of infection; then IgG levels rose later. It exhibited the risk of a false-negative diagnosis of RT-PCR in COVID-19 testing. The significant detection ability of this new optical-based platform demonstrated clinical potential.

## Introduction

The coronavirus disease (COVID-19) is the global public health challenge currently persisting at a grand scale. It first presented a local outbreak of pneumonia of initially unknown pathogens in Wuhan (Hubei, China) in December 2019 [1]. The whole-genome sequence of COVID-19 was quickly identified based on the genetic similarity to bat SARS-CoV-like coronaviruses, and multiple vaccine candidates are currently undergoing phase 3 clinical trials. Accordingly, rapid screening and strict quarantine measures have become pivotal approaches for limiting the spread of the COVID-19 pandemic.

Since the COVID-19 pandemic outbreak, the effectiveness of screening tests is one of the critical issues. To contain the pandemic, fast and reliable tests for COVID-19 are in urgent need worldwide. Reverse transcriptase-polymerase chain reaction (RT-PCR) and IgM/IgG enzyme-linked immunosorbent assay (ELISA) are the two major test types for diagnosing viral infections [2, 3]. RT-PCR remains the gold standard test for diagnosing current and past COVID-19, which directly detects the pathogen’s RNA [4]. Despite this, RT-PCR’s detection of viral nucleic acid requires sufficient quantities of high-quality viral RNA, which is sometimes difficult to obtain due to variances in sampling technique, patient viral load, the timing of infection, and the onset of symptoms.

Serological tests were primarily employed to aid nucleic acid tests in the past, providing an extended window for detecting active and recent infections. Yet, with the advancement of vaccine developments, these serological tests possess another crucial function: determining changes in the recipient’s antibody level before and after vaccination. Meanwhile, the US Food and Drug Administration (FDA) has urgently approved an emergency use authorization (EUA) for plasma therapy [5]. Plasma therapy is primarily adopted to obtain convalescent plasma from recovered COVID-19 patients, thereby treating COVID-19 through the presence of antibodies against the virus in the plasma [6]. Rapid antibody quantitative test is essential for plasma therapy. The FDA also announced that priority would be given to reviewing products that can improve the availability of testing and significantly enhance testing capabilities, hoping to reduce dependence on laboratory testing [7].

At present, further improvement of the accuracy of test reagents on the market can be achieved, with most reagents lacking the quantitative ability of COVID-19 antibodies. Quantitative knowledge is beneficial for determining the current phase of infection of a patient. It is necessary to have a method that meets the rapid quantitative detection of antibodies to assess the body’s immune response from natural COVID-19 illness or vaccines’ effects. Here, a simple platform that could be easily performed outside the laboratory would be beneficial. For this reason, a Lateral flow immunoassay (LFIA) coupled with a portable spectrometer (Spectrometer +ACE Biolabs COVID-19 IgG / IgM Dual Detection Kit) was developed.

Further, an attempt was made to integrate a newly designed spectrometer to the COVID-19 test strip procedure; this augmentation provides the quantitative capacity to a LFIA. This detection method can quickly produce results in 5–10 minutes. The developed product was compared with several other serological IgM/IgG antibody reagents on the market, hoping to arrive at a relatively objective conclusion.

## Methods

### Patients and samples

The present study was approved by the Joint Institutional Review Board (JIRB) of the Taipei Hospital, Ministry of Health and Welfare (IRB numbers: TH-IRB-0020-0011). The present study was conducted following the relevant regulations and guidelines. 111 subjects aged 20–79 years old suspected of having COVID-19 infection (e.g., fever ≥38°C or acute respiratory symptoms) were recruited from a hospital in Taipei from March to May 2020. All study subjects have provided informed consent. Participants completed a survey questionnaire that includes demographic information: birth year, travel, contact, cluster, education levels, occupation, family income, alcohol, drug use, and lifestyle habits. Clinical symptoms such as cough, sore throat, general soreness, etc., were also collected. In addition, blood samples and swab specimens were collected at the hospital. Patients suspected of having COVID-19 disease premised on relevant medical history and symptoms were included in this study. The exclusion criteria included (1) Participants who had nasal or oral structure problems for sampling (such as oral cancer). (2) The participant was deemed unsuitable due to the present illness (such as undergoing Cardiopulmonary Resuscitation). Table 1 illustrates the demographic characteristics of all participants.

**Table 1:** Essential demographic characteristics of participants.

Characteristics	Confirmed, PCR(+)	Suspected, PCR(-)	P-value
	(N = 12)	(N = 99)	
Age (mean ± SD)	28.00±11.68	37.17±19.59	0.116
Male	6 (50.0%)	49 (49.5%)	0.974
Symptom			
Fever	6 (50.0%)	55 (55.6%)	0.765
Cough	4 (33.3%)	43 (43.4%)	0.504
Sore throat	4 (33.3%)	18 (18.2%)	0.214
Diarrhea	1 (8.3%)	13 (13.1%)	0.636

Nasopharyngeal or oropharyngeal swab specimen was collected from each patient and sent for Reverse transcriptase-polymerase chain reaction (RT-PCR) testing. Blood samples of patients were collected on 0–30 days after symptom onset and examined by the LFIA.

### Laboratory method

The RT- PCR was performed by LabTurboTM AIO SP-qPCR Dual-Panel Automation System. It uses one SARS-CoV-2 specific primer pair and probe that explicitly recognizes the SARS-CoV-2 RNA sequence (N1 gene) and is designed to achieve high sensitivity (LOD 20 copies/ml) the examination.

Relying on immunochromatography principles, all COVID-19 rapid test strips in the present study used colloidal gold-conjugated antibodies to detect human IgG (Immunoglobulin G) and IgM (Immunoglobulin M) antibodies. A nitrocellulose strip was pre-coated with perpendicular recombinant viral COVID-19 protein lines that could capture reagents in this device. As the applied sample flowed down the strip, anti-COVID-19 IgG and IgM antibodies would form colloidal gold antibody-conjugated IgG and IgM complexes. The perpendicular IgG and IgM lines of the test strip captured these complexes, upon which colored bands would be presented. Moreover, although antibody levels vary between individuals, in most cases, the IgM is potentially helpful for detecting recent infection, which usually becomes undetectable in the weeks following infection. In contrast, the presence of IgG antibodies often indicates a past infection as it generally does not appear until 7 to 10 days and may last for months or years after infection.

**Fig 1:**
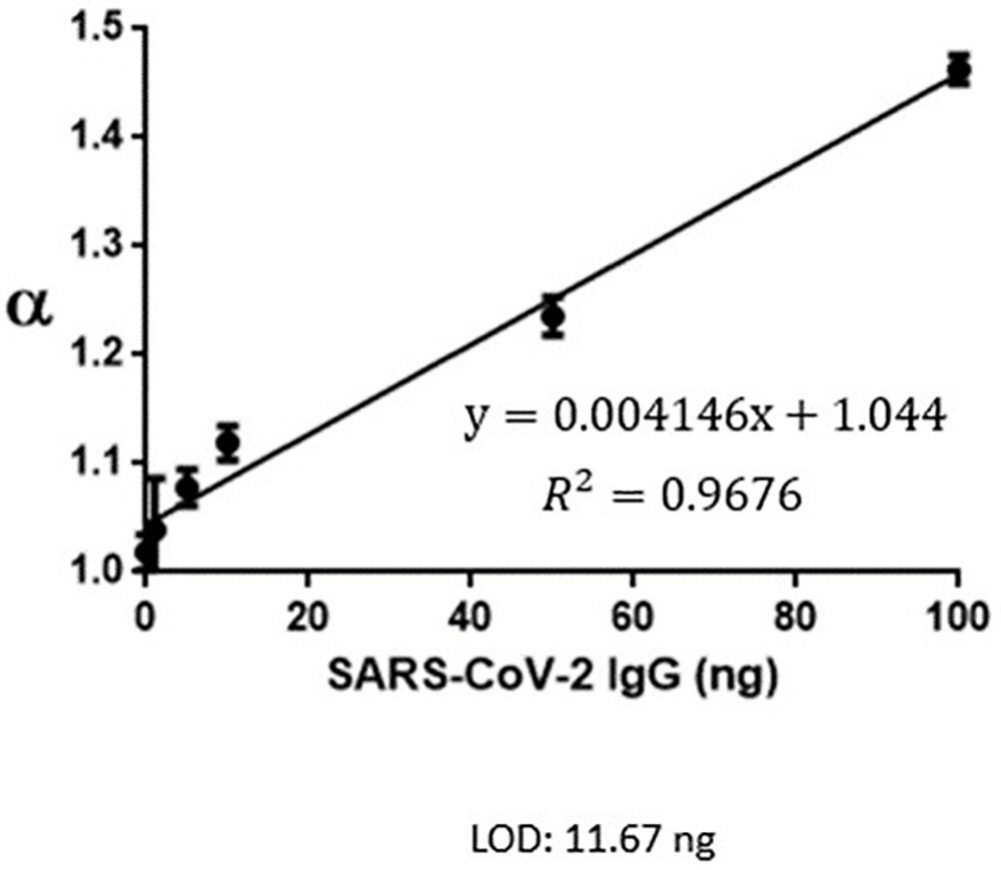
SARS-CoV-2 IgG standard curve.

**Fig 2:**
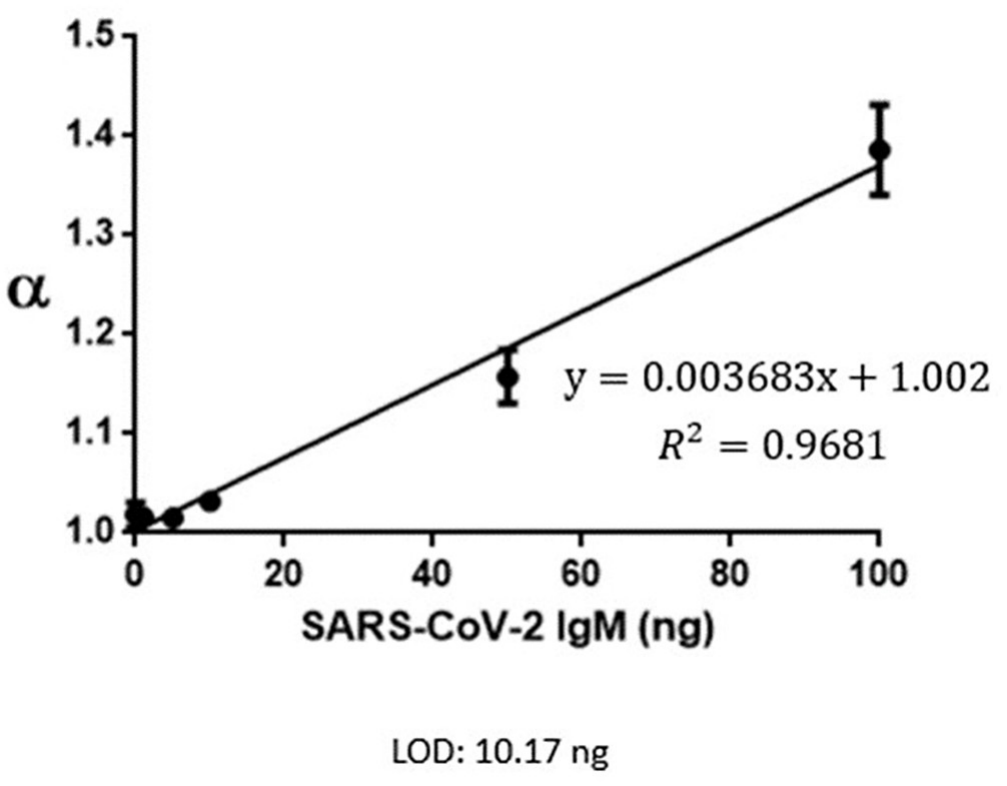
SARS-CoV-2 IgM standard curve.

LFIA is a paper-based in situ detection platform that is characteristically inexpensive, easy to use, and operated by a non-healthcare provider. While LFIA is an ideal point-of-care test, the conventional colorimetric LFIA has an unsatisfactory limit of detection (LOD). Biochemical manufacturers provide us with different concentrations of standard samples. We obtain the value (α) by spectrometer system then establish a standard curve of IgG (Fig 1) and IgM (Fig 2). We set cutoff threshold (α = 1.05), LOD α(IgG): 11.67ng; LOD α(IgM): 10.17ng.


$$\text{LOD}=3\times \text{Blank}\left(\text{standard}\;\text{deviation}\right)/\text{Slope}\;\text{of}\;\text{standard}\;\text{curve}$$

Then we used the LOD formula to calculate the SARS-CoV-2 IgG and IgM detection limit, respectively, where reflectance value is converted from the raw spectrum. The content of our article will explain in detail how to learn positive and negative thresholds. The cutoff threshold (α = is at 1.05) is set with COVID-19 clinical positive and negative sera. LOD spiking test and clinical α cutoff test are two independent experiments. The subtle color variations are difficult to differentiate visually (Table 2). Therefore, we use the spectrometer platform to improve sensitivity and specificity.

**Table 2:** Lateral flow immunoassays for COVID-19 diagnostic testing.

Assay	BioMedomics	ACE Biolabs	ASK	TBG	Spectrometer + ACE Biolab
**Assay principle**	LFIA	LFIA	LFIA	LFIA	Spectra reader + LFIA(N+S)
**Solid-phase antigen**	Nucleocapsid (N)	N+S	Spike (S)	N+S	N+S
**Specimen type**	Whole blood, serum, plasma	Whole blood, serum, plasma	Whole blood, serum, plasma	Whole blood, serum, plasma	Serum, plasma
**Certified state**	CE	FDA EUA / TFDA Submitted	TFDA	FDA EUA / CE	1. TFDA/FDA 510(k) E
				TFDA Submitted	2. FDA EUA / TFDA Submitted
**Result calculation**	Visual	Visual	Visual	Visual	Index(α) = 1.05
	Qualitative	Qualitative	Qualitative	Qualitative	Quantitative
**Positive Cutoff Threshold**	10ng/mL	5ng/mL	NA	NA	0.5ng/ml (Reader)
**Reported performance**	Sensitivity = 100%	Sensitivity = 96%	Sensitivity = 73%	Sensitivity = 93%	-
	Specificity = 98%	Specificity = 100%	Specificity = 100%	Specificity = 95%	
**Specimen amount**	10–20 uL	10–20 uL	10–20 uL	10–20 uL	10–20 uL
**Turnaround time**	15 min	10–15 min	10 min	10–15 min	5–10 min

Abbreviations: BioMedomics, COVID-19 IgM/IgG Rapid Test (BIOMEDOMICS Inc.); ACE Biolabs, COVID-19 IgG / IgM Dual Detection Kit (ACE Biolabs Inc.); ASK, COVID-19 Antibody Rapid Teat (TONYAR BIOTECH Inc.); TBG, SARS-CoV-2 IgG/IgM Rapid Test Kit (TBG Diagnostics Limited.); LFIA, *Lateral flow immunoassay*; N, Nucleocapsid; S, Spike; TFDA, *Taiwan Food and Drug Administration*; FDA, US *Food and Drug Administration; EUA*, Emergency Use Authorization; CE, Communate Europpene.

**Fig 3:**
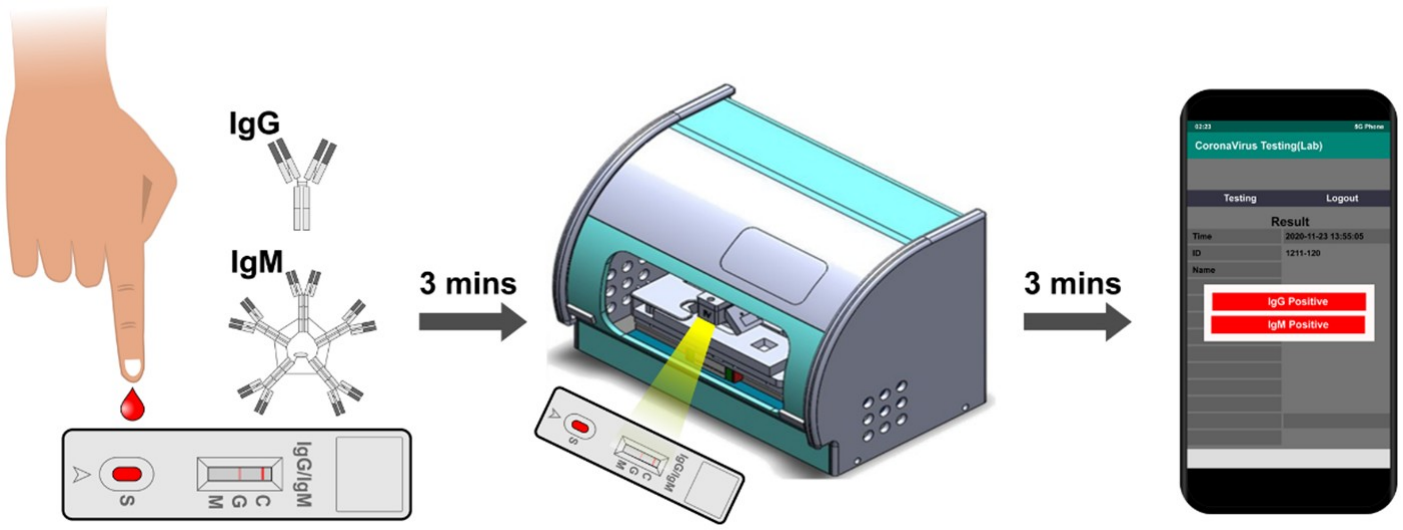
The workflow of quantitative spectral LFIA platform. The optical-based platform applies 10–20 uL of blood from a fingertip or a vein to the test strip, integrating the immunoglobulins and producing results in 5–10 minutes.

The chromophores on paper test strips emit complex spectra at various wavelengths that a spectrometer can readily detect to provide precise and quantifiable information. Hence, coupling the LFIA with the portable spectrometer improves sensitivity and enables quantitative analysis (Table 2 with gray background). Also, when a gold colloid is employed, a suspension consisting of sub-micron gold nanoparticles dissolves insolvent can enhance the visibility and stability of the LFIA. It is believed that the increased detection limit using this method could render this new platform an invaluable tool for accurate serological COVID-19 antibody testing. The spectra immune rapid analyzer applies 10–20 uL of blood from a fingertip or a vein to the test strip and can provide results quickly, as in 5–10 minutes (Fig 3).

**Fig 4:**
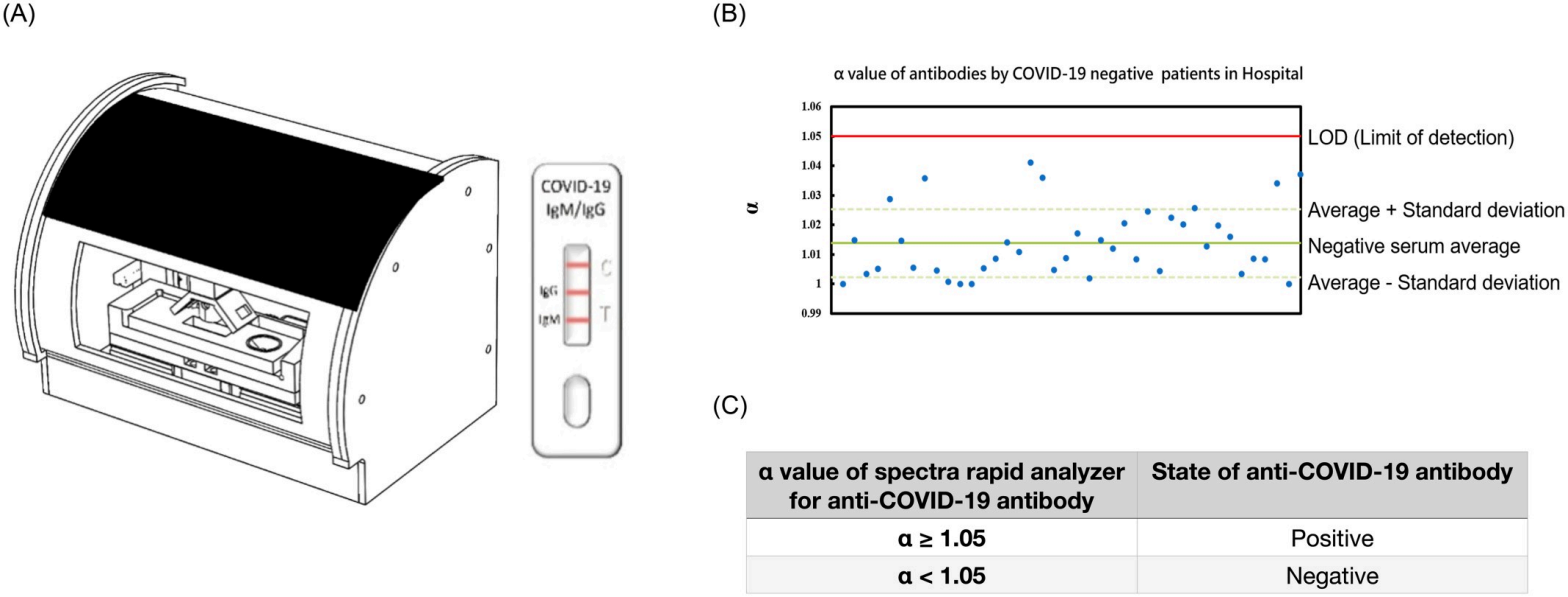
The platform learned LOD (Limit of detection) from the database of 40 COVID-19 negative patients. Lateral flow immunoassay formed colloidal gold antibody-conjugated IgG/IgM complexes on the colored bands. Then, LOD was determined by α value, which refers to the color reflection value of the antibody-conjugated IgG/IgM complexes by optical scanning. Finally, specimens of 40 COVID-19 negative patients were employed to establish the standard LOD (limit of detection) interval.

Examiners could perform quantitative spectral analysis by using a spectrometer coupled with a test strip. An app of smartphone activated the automatic scanning of the rapid test strip. Full-spectrum antibody reflex optical signals were acquired from the spectral optical module to analyze COVID-19 IgG/IgM full-spectrum antibody distribution and concentration with standard quantification. The results can be used in conjunction with clinical timetables to analyze and track the spread of COVID-19. The quantitative spectral LFIA platform (SpectroChip Inc.) detects IgM/IgG antibodies from test kits (Fig 4).

Designed for use with a LFIA COVID-19 IgG rapid test cassette, this spectra reader provides high resolution (3–5 nm) results across a vast spectral range (300 to 1000 nm). The quantitative spectral LFIA uses immunoassay technology and the principle of chromatography to fix specific antibodies or antigens on nano-grade colloidal gold particles(Colloidal gold) as a reagent coloring agent to detect whether there is an immune response in the specimen. The platform detects the antibody test strip’s reflectance spectrum, where the primary reflectance wavelengths are minimum wavelength, and the central reference wavelength is 650 nm. The α value was calculated via ranges at minimum reflectance and reference wavelengths:

$$\alpha =\text{Reflectance}\;\left(\text{at}\;650\;\text{nm}\right)/\text{Reflectance}\left(\text{Minimum}\right)$$

where α value refers to the color reflection value of the optical scanning antibody rapid screening test piece. After forming colloidal gold antibody-conjugated IgG and IgM complexes, the color depth reflects the antibody concentration. A higher α value indicates a stronger reflection color intensity, which infers higher antibody concentration. Specimens of 40 COVID-19 negative patients were employed to establish the standard LOD interval for judging a patient as a positive or negative infection. The COVID-19 negative patient sample signal was used to calibrate the test, with the calculated signal to cutoff (α) value of <1.05 and ≥1.05 to report them as negative and positive, respectively. The spectrum data of the patients exceeding the detection limit zone was considered positive.

### Statistical analysis

Data were expressed as mean ± standard deviation for normally distributed, continuous variables and proportions for categorical variables. Continuous variables were analyzed utilizing a two-tailed t-test, while discrete variables were compared using a Chi-square test. The difference in sensitivity or specificity of the antibody test was compared with RT-PCR through the McNemar test. A *p*-value of <0.05 was considered statistically significant. Statistical analysis was executed using SAS (version 9.4.; SAS Institute Inc., Cary, NC, USA).

## Results

RT-PCR test confirmed 12 PCR-positive patients and 99 PCR-negative patients. There was no significant difference in demographic characteristics and symptoms between groups (Table 1).

The performance of the five types of LFIA was highlighted in Table 3. We chose ACE COVID-19 IgG / IgM Dual Detection Kit (ACE Biolabs), TBG SARS-CoV-2 IgG/IgM Rapid Test Kit (TBG Diagnostics Limited.), ASK COVID-19 Antibody Rapid Teat (TONYAR BIOTECH Inc.), and BioMedomics COVID-19 IgM/IgG Rapid Test (BIOMEDOMICS Inc.) for comparison. Among the 12 RT-PCR positive patients, the LFIA test result of BioMedomics, ACE, ASK, and TBG was positive in 7 (58.33%), 11 (91.67%), 6 (50%), and 10 (83.33%) patients, respectively. The detection capability of Spectrometer +ACE Biolabs was 12 (100%).

**Table 3:** The performance of 5 types of lateral flow immunoassays.

	Sample from RT-PCR result		Sample from days after symptom onset (Total = 12)			
	Positive (N = 12)	Negative (N = 99)	0–4 days (N = 5)	5–8 days (N = 2)	9–13 days (N = 3)	≥14 days (N = 2)
**BioMedomics**	58.33% (7/12)	95.96% (95/99)	40% (2/5)	0% (0/2)	100% (3/3)	100% (2/2)
**ACE Biolab**	91.67% (11/12)	96.97% (96/99)	80% (4/5)	100% (2/2)	100% (3/3)	100% (2/2)
**ASK**	50% (6/12)	-	40% (2/5)	0% (0/2)	33.33% (1/3)	100% (2/2)
**TBG**	83.33% (10/12)	-	60% (3/5)	100% (2/2)	100% (3/3)	100% (2/2)
**Spectrometer + ACE Biolab**	100% (12/12)	97.98% (97/99)	100% (5/5)	100% (2/2)	100% (3/3)	100% (2/2)

Abbreviations: BioMedomics, COVID-19 IgM/IgG Rapid Test (BIOMEDOMICS Inc.); ACE Biolabs, COVID-19 IgG / IgM Dual Detection Kit (ACE Biolabs Inc.); ASK, COVID-19 Antibody Rapid Teat (TONYAR BIOTECH Inc.); TBG, SARS-CoV-2 IgG/IgM Rapid Test Kit (TBG Diagnostics Limited.)

The sensitivity of the other four assays increased after nine days of symptoms onset. (range, 33% to 100%). All assays achieved 100% sensitivity in samples collected after at least 14 days following initial symptoms. The ACE and TBG assay sensitivities were 91.67% (11/12) and 83.33% (10/12) for 12 positive samples collected 0–30 days post-symptom onset. However, the one false-negative blood sample in the ACE and TBG tests was caused by a hemolysis of specimen in this patient (the specimen was collected on the third day of symptoms). By taking RT-PCR as the gold standard of diagnosis, the overall sensitivity and specificity of LFIA could be determined via blood samples collected for one-month duration after symptoms onset.

Among the 99 RT-PCR negative patients, the BioMedomics and ACE Biolabs anti-COVID-19 IgM and IgG assays were negative in 95.96% (95/99) and 97.98% (96/99) of samples, respectively (Table 3).

The quantitative spectral LFIA platform (Spectrometer +ACE Biolabs) employed the high sensitivity LFIA of ACE to provide precise assay results. The assay was quantitative in design, and the mean α value set for COVID-19 positive patients was over the LOD in any convalescent-phase sample with 100% sensitivity (see Table 3). Confidence intervals for sensitivity and specificity were calculated using a PPA score method (Positive Percent Agreement) and NPA (Negative Percent Agreement). The test was validated against a panel of previously frozen samples consisting of 12 Covid-19 positive blood samples and 99 Covid-19 negative blood samples. Each of the 12 Covid-19 positive pieces was verified with a nucleic acid amplification test, and both IgM and IgG antibodies were ensured to be present in all 12 samples. All Covid-19 negative samples (n = 99) were collected and compared to COVID-19 PCR positive status. Clinical results and summary statistics are reported in Table 4.

**Table 4:** COVID-19 IgG/IgM antibody rapid tests.

Venous whole blood			RT-PCR Test		Total
			POS	NEG	
**ACE BioLabs**	**POS**	**IgM+/IgG+**	**3**	**1**	**14**
		**IgM+/IgG-**	**1**	**0**	
		**IgM-/IgG+**	**7**	**2**	
	**NEG**	**IgM-/IgG-**	**1**	**96**	**97**
	**Total**		**12**	**99**	**111**
	**Sensitivity 92% (11/12)**				
	**Specificity 97% (96/99)**				
**Bio Medomics**	**POS**	**IgM+/IgG+**	**1**	**1**	**11**
		**IgM+/IgG-**	**0**	**0**	
		**IgM-/IgG+**	**6**	**3**	
	**NEG**	**IgM-/IgG-**	**5**	**95**	**100**
	**Total**		**12**	**99**	**111**
	**Sensitivity 58% (7/12)**				
	**Specificity 96%(95/99)**				
**Spectrometer + ACE BioLabs**	**POS**	**IgM+/IgG+**	**3**	**1**	**14**
		**IgM+/IgG-**	**2**	**0**	
		**IgM-/IgG+**	**7**	**1**	
	**NEG**	**IgM-/IgG-**	**0**	**97**	**97**
	**Total**		**12**	**99**	**111**
	**Sensitivity 100% (12/12)**				
	**Specificity 98%(97/99)**				

Abbreviations: BioMedomics, COVID-19 IgM/IgG Rapid Test (BIOMEDOMICS Inc.); ACE Biolabs, COVID-19 IgG / IgM Dual Detection Kit (ACE Biolabs Inc.)

**Fig 5:**
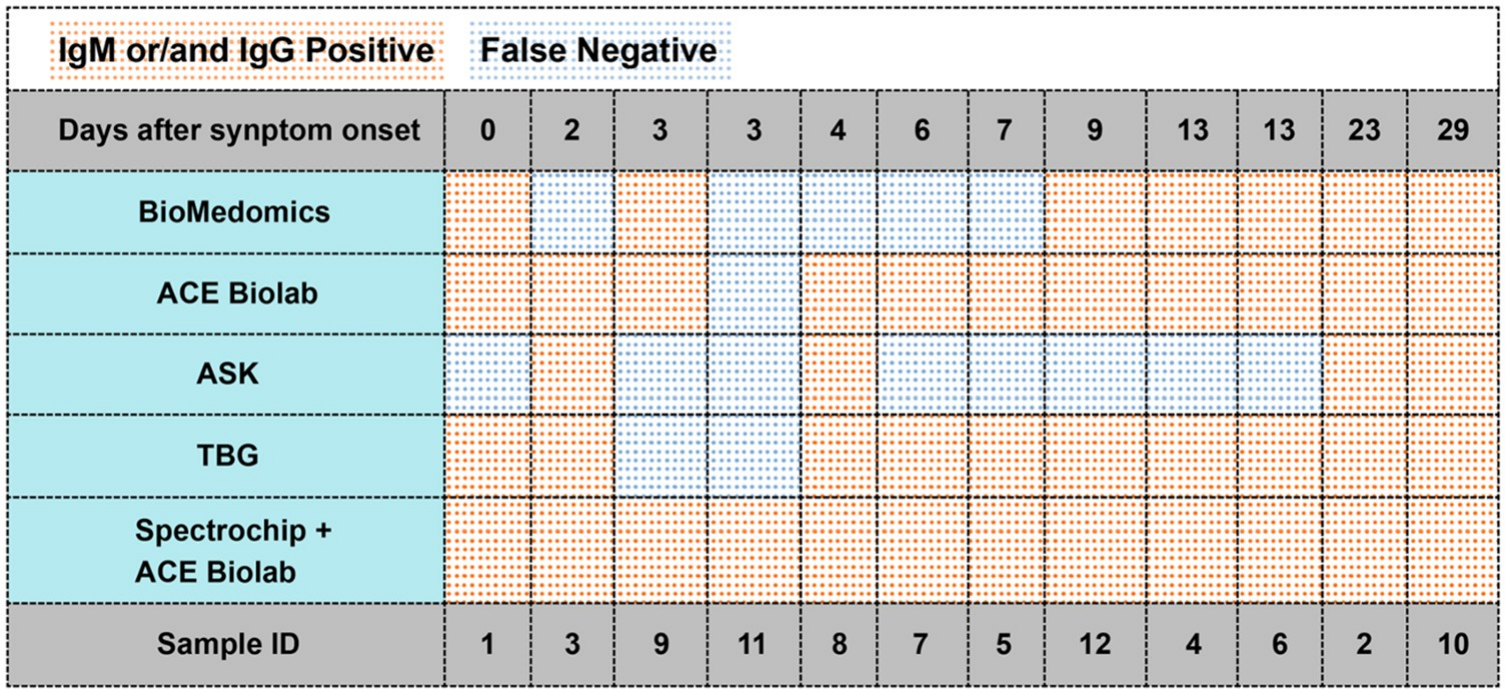
The performance of five types of lateral flow immunoassays. Samples of 12 RT-PCR positive subjects were followed up by days after symptoms onset. Abbreviations: BioMedomics, COVID-19 IgM/IgG Rapid Test (BIOMEDOMICS INC.); ACE Biolabs, COVID-19 IgG / IgM Dual Detection Kit (ACE Biolabs INC.); ASK, COVID-19 Antibody Rapid Teat (TONYAR BIOTECH INC.); TBG, SARS-CoV-2 IgG/IgM Rapid Test Kit (TBG Diagnostics Limited.).

The platform’s sensitivity was good in samples collected 0 to 4 days post-symptom onset (range, 80% to 100%). Further, 100% sensitivity was achieved in samples collected for a one-month duration following initial disease manifestation. The P-value showed a significant difference between COVID-19 negative and COVID-19 positive patients (p<0.001). The temporal kinetics of the anti-COVID-19 antibody response of COVID-19 positive patients for each of the five evaluated assays and spectra analyzer was exhibited (Fig 5), both inpatients that seroconverted during their hospital stay and those who had at least one serial draw available (n = 12).

**Fig 6:**
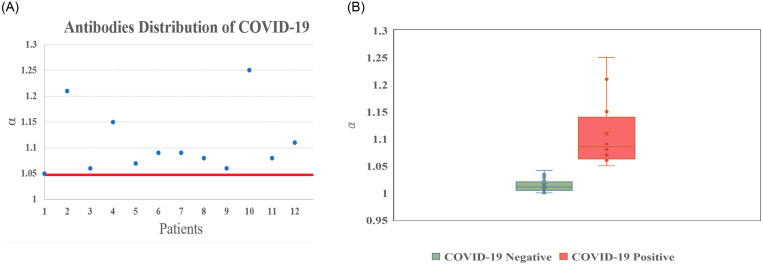
The α value in different groups of Covid-19 diagnosis. The quantitative optical-based LFIA platform showed a significant difference of α value in COVID-19 RT-PCR negative and positive patient groups.

The LFIA of ACE had the lowest COVID-19 screening false negative in the early stage of infection. In addition, the spectra analyzer of the spectrometer improved the lower limit of detecting immunoglobulins (Fig 6).

**Fig 7:**
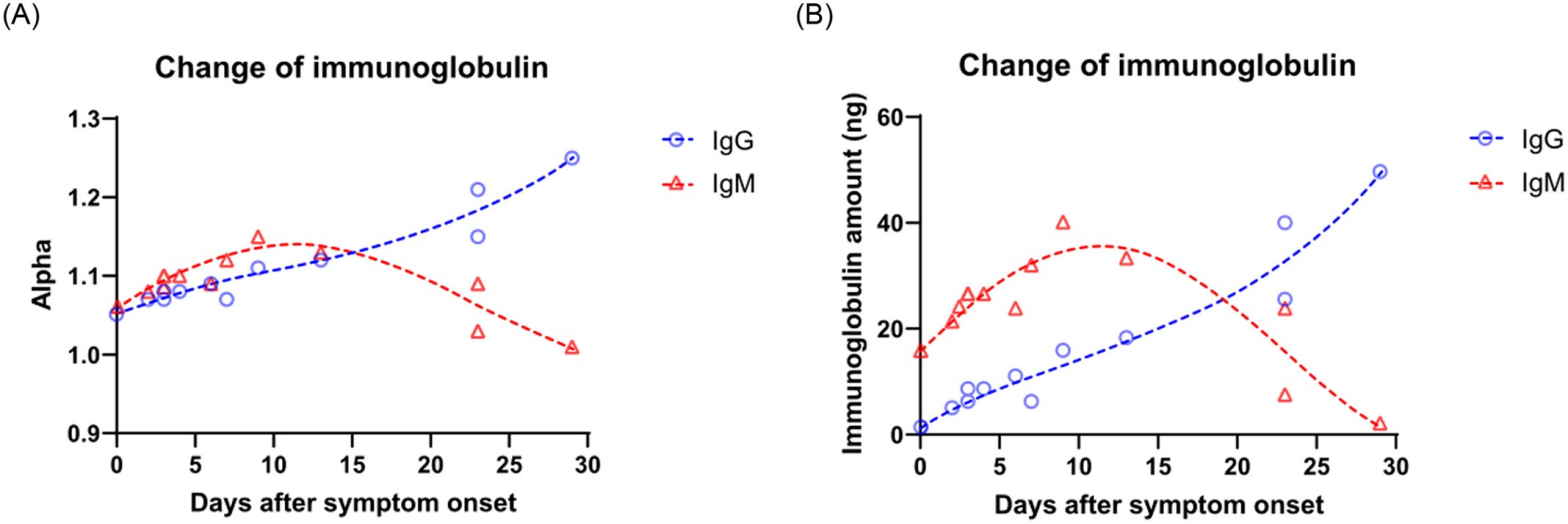
Increase of COVID-19 antibodies distribution by days after symptoms onset. After converting the α value into the actual antibody concentration unit (ng), a trend could be observed in the Covid-19 RT-PCR positive group that IgM reached the peak value first, and then IgG rose and maintained.

As shown in Table 4, the different COVID-19 IgG and IgM antibody results were presented. IgM is an antibody that belongs to the acute reaction phase, while IgG is related to long-term immune protection. It illustrates an apparent trend that elevation of IgM levels in the acute phase of infection; then IgG levels rose later (Fig 7). The spectral LFIA platform employed a defined immune response for the COVID-19 infection period. After that, the IgM antibody concentration increases and reaches higher levels by 7–10 days and almost disappears by 28–30 days, wheres protection IgG antibody rise in 5–10 days and persist.

## Discussion

The analytical platform of Spectrometer +ACE Biolabs can attain a detection limit of 0.362 ng/mL for the human anti-spike protein IgG/IgM, with only a drop of blood needed to produce a result in 5–10 minutes. It is beneficial in assessing the patient’s antibody levels after vaccination or infection and can play an essential role in clinical practice.

In Table 3, if RT-PCR positive is taken as the diagnostic gold standard, the analytical platform (Spectrometer +ACE Biolabs) could correctly detect all 12 COVID-19 patients (100% sensitivity, 12/12). Conversely, the sensitivity for ACE Biolabs was 91.67% (11/12), while BioMedomics was 58.33% (7/12). Additionally, we found that methods that use Nucleocapsid (N) + Spike (S) solid-phase antigen (i.e., ACE Biolabs, TBG, and Spectrometer +ACE Biolabs) perform better, compared with those that use Nucleocapsid (BioMedomics) or Spike (ASK) alone. In the 12 patients with a confirmed positive diagnosis for COVID-19 by RT-PCR, several antibody reagents still presented as negative 13 days after the onset of symptoms. It could be attributed to the following two situations: 1) The level of antibodies in the early stages was too low, whereby the LOD of some test reagents was unable to detect the minimal amounts of antibodies presence; and 2) There may be errors that occurred during sample collection or machine operation.

About RT-PCR negative patients, all three antibody testing methods found positive cases. One explanation is that there may have been patients who have been infected with COVID-19 that were missed by the RT-PCR test. In most patients, viral RNA often becomes detectable as early as day one and peaks within the first week of symptom onset. To be specific, the detection of viral nucleic acid by RT-PCR requires sufficient quantities of high-quality viral RNA, which is sometimes difficult to obtain due to variances in sampling technique, patient viral load, the timing of infection, and the onset of symptoms. Further, RNA samples are vulnerable to degradation. Well-trained scientists are required to perform PCR utilizing complex laboratory equipment [8]. Simultaneously, the transportation to the laboratory and the requirement to batch multitudinous samples also limit RT-PCR application. Studies have highlighted that RT-PCR in COVID-19 testing exhibits up to 41% false-negative diagnosis. Yet, the existence of such false-negative RT-PCR results still requires further research [9–11].

Simultaneously, there is a scarcity of information regarding the detection limit of serological assays than RT-PCR. Notably, serology testing may overcome some limitations of RT-PCR. In rare situations where RT-PCR is not possible, serological testing could be adopted. Phlebotomy is less technique-sensitive and produces minor sample variance. Blood antibodies are more stable than viral RNA collected by swabbing, and blood can be readily kept and tested in a device. A non-healthcare provider is often capable of operating a serology test [12, 13].

The FDA implied that a negative RT-PCR test result does not wholly exclude COVID-19 infection. Thus, healthcare providers should not treat RT-PCR as a single element for diagnosis, and re-taking a test is reasonable in the presence of any doubt [14]. An additional serological antibody test could play a supporting role.

Most reports of COVID-19 of antibody reaction are skewed toward severe and hospitalized cases. In asymptomatic and presymptomatic patients, the detection effectiveness is not so clear. Notably, 10 out of 175 PCR-confirmed patients in one study had mild symptoms of COVID-19 and eventually did not develop antibodies, raising concerns regarding the use of serological antibody tests for screening [15]. A likely assumption is that the magnitude of antibody reaction was not detectable via currently available serological tests. Yet, the false-positive of RT-PCR results should remain a concern. Immune delayed reaction of COVID-19 is another limitation of the serological antibody test. The targets of serological assays, primarily IgM or IgG antibodies, often increase 7 to 10 days after symptoms [2]. Most patients tested positive for antiviral IgG within 14 to 19 days after symptom onset [16–18]. In early infection, the immune response is evolving, and viral-specific antibodies are potentially too minimal to be detected using current serological test methods [19]. It’s challenging to identify low-concentration gold nanoparticles just with the naked eye by current commercial kit protocol. A susceptible spectrometer system integrated with a high magnification optical lens is used to measure the reflectance spectrum of the gold nanoparticles. This integrated spectrometer system is so sensitive that it can detect the minimal amount of the gold nanoparticles within 0–4 days of disease onset. Accordingly, an improved ELISA-based assay, preferably a LFIA considering the readiness for deployment and use by the general public thereof, may provide an adjective option in COVID-19 pandemic.

The platform adopted in the present research can produce results in just 5–10 minutes without a blood draw. It is much faster than RT-PCR, which takes about 1.5 hours to run an exam. Detection could be conducted via just a drop of blood, increasing people’s willingness to cooperate and reduce errors caused by the sampling of the throat or nasopharyngeal swabs. Besides, the detailed IgM and IgG values would reveal the disease’s progress. The serological antibody tests could fill up the missing gap of RT-PCR as an auxiliary role in the Covid-19 pandemic. Moreover, in remote areas where performing RT-PCR is inconvenient, rapid antibody screening is also advantageous.

In the early stages of infection, the reagent bars representing positive results are not apparent because of insufficient antibodies. Therefore, the LOD would be inferior, with only the naked eye being used to judge the outcome (Visual Qualitative). For this reason, machine detection of the reagent result by α value was developed. The machine adopted in the present article can be used for both antibody and antigen detection. However, the antibody exam function was chosen for comparing the ability with other rapid serology antibody screening reagents.

The lack of positive cases is one of the limitations of our study. However, statistical significance was achieved, and the usefulness and accuracy of the test were demonstrated. The prevalence of Covid-19 positive cases was scarce in Taiwan when it was the initial outbreak. In terms of the newly developed test method, fewer patient numbers were inevitable since this method was not promoted. Meaningful improvement of sensitivity and specificity was observed after augmentation of the spectrometer to the LFIA.

The is another limitation of spectrum bias. We assumed that sensitivity was highest for severe infections and considered a range of values of asymptomatic and mild infections [20]. The subjects of our study are mainly patients who were hospitalized due to pneumonia. Therefore, it is representative of this group of sicker patients. However, if it is used in public for general screening, the effectiveness of these quick screening reagents may be somewhat overestimated in this study.

This LFIA, coupled with a portable spectrometer, demonstrated improved sensitivity and enabled quantitative analysis. The present research is predicated on objective comparisons with similar serological immune assays on the market. Different periods after diagnosis were tracked to reveal the changes in results due to variation in antibody levels.

## Conclusion

This study’s platform showed high sensitivity and specificity in detecting trace amounts of COVID-19 antibody IgG/IgM. Optical interpretation (rather than visual qualification) of results by quantitative α index improved the accuracy. Both Nucleocapsid (N) + Spike (S) solid-phase antigen detection also increased the sensitivity. This quick screening test could accurately measure antibody levels at the very beginning of the disease. It could be a useful evaluation tool for evaluating the natural course of the illness or antibody response from vaccines for COVID-19.

## Supporting information

S1 File Coronavirus (Covid-19) health survey questionnaire. We use this questionnaire to evaluate patients’ basic information, contact history, and symptoms associated with Covid-19.
(PDF)
