# Supplementary material for: The early detection of immunoglobulins via optical-based lateral flow immunoassay platform in COVID-19 pandemic
Source: PLoS One. 2021 Jul 20;16(7):e0254486. doi: 10.1371/journal.pone.0254486 (PMC8291656; doi:10.1371/journal.pone.0254486)
Supplement: S1 File — We use this questionnaire to evaluate patients’ basic information, contact history, and symptoms associated with Covid-19. (PDF) [file pone.0254486.s001.pdf]

Coronavirus (Covid-19) Health Survey Questionnaire

Name: ______________________

Age: ______________________

Gender: ______________________

Date: ______________________

Have you travelled to outbreak areas of the coronavirus disease in the past 14 days?	☐ YES ☐ NO
Are you currently required to be in isolation because you have been diagnosed with coronavirus (COVID-19)?	☐ YES ☐ NO
Are you a healthcare worker?	☐ YES ☐ NO
Did you have any contact with patients confirmed with coronavirus disease in the past 14 days?	☐ YES ☐ NO
Have you been directed to a period of 14-day quarantine by the Taiwan Ministry of Health and Welfare as a result of being a close contact of someone with coronavirus (COVID-19)?	☐ YES ☐ NO

Are you experiencing these symptoms?

Fever (You are considered to have a fever if body temperature is above 37.5°C)	☐ YES ☐ NO
Chills	☐ YES ☐ NO
Cough	☐ YES ☐ NO
Sore throat	☐ YES ☐ NO
Shortness of breath	☐ YES ☐ NO
Runny nose	☐ YES ☐ NO
Loss of sense of smell	☐ YES ☐ NO
Conjunctivitis (Pink eye)	☐ YES ☐ NO
Diarrhea	☐ YES ☐ NO

Do any of your family members or close contacts also suffer from the same symptoms?	☐ YES ☐ NO

新冠病毒 (Covid-19) 疫情健康調查表

姓名: ______________________

年齡: ______________________

性別: ______________________

日期: ______________________

在過去的 14 天裡，您有沒有去過冠狀病毒的流行地區？	☐ 是 ☐ 否
您當前是否被診斷出患有冠狀病毒（COVID-19）而需要接受隔離？	☐ 是 ☐ 否
您是醫護人員嗎？	☐ 是 ☐ 否
在過去的 14 天內，您是否與確診冠狀病毒的患者有過接觸？	☐ 是 ☐ 否
您是否由於與冠狀病毒（COVID-19）的接觸，而被台灣衛生福利部指定為期 14 天的隔離期？	☐ 是 ☐ 否

您是否有過這些症狀？

發燒（體溫高於 37.5˚C 時，視為發燒）	☐ 是	☐ 否
畏寒	☐ 是	☐ 否
咳嗽	☐ 是	☐ 否
咽喉痛	☐ 是	☐ 否
喘	☐ 是	☐ 否
流鼻涕	☐ 是	☐ 否
嗅覺喪失	☐ 是	☐ 否
結膜炎（紅眼睛）	☐ 是	☐ 否
腹瀉	☐ 是	☐ 否

您的家人或朋友是否也有同樣的症狀？	☐ 是 ☐ 否
